# TERT and its binding protein: overexpression of GABPA/B in high grade gliomas

**DOI:** 10.18632/oncotarget.27985

**Published:** 2021-06-22

**Authors:** Efthymios Papazacharias, Saskia Kuhl, Gabriele Röhn, Lukas Görtz, Roland Goldbrunner, Marco Timmer

**Affiliations:** ^1^Laboratory of Neurooncology and Experimental Neurosurgery, Department of General Neurosurgery, Center for Neurosurgery, Faculty of Medicine and University Hospital, University of Cologne, Cologne, Germany

**Keywords:** TERT, GABPA, GABPB, glioma, astrocytoma

## Abstract

Enhanced expression of TERT in gliomas is a result of two hotspot mutations, C228T and C250T, at the promoter region. GA-binding proteins selectively bind at these positions, respectively, causing an activation of the promoter and overexpression of TERT. GABP is a multimeric protein consisting of GABPA and GABPB with its isoforms GABPB1, GABPB1-L, GABPB1-S, GABPB2.

In this study, we investigated the mRNA expression and association between TERT and GABPA/B isoforms in tumor samples of different glioma grades. The expression was determined by quantitative real-time PCR and the results were statistically analyzed.

We present that TERT is mainly expressed in primary glioblastomas. All GA-binding proteins progress through the glioma grades and have the highest expression levels in secondary glioblastomas. In secondary glioblastomas after chemotherapy, GABPB1 and GABPB1-L are expressed on a lower level than without treatment. In high grades, TERT and GABPA, GAPB1, GABPB1-L, GABPB1-S are upregulated compared to low grades. Between primary and secondary glioblastomas with and without chemotherapy, TERT is elevated in the former while GABPB1 is increased in the secondary glioblastomas. GABPA and GABPB1, GABPB1-L and GABPB1-S positive correlate in primary glioblastomas.

The present study confirms the upregulation of TERT in primary glioblastomas while all GABP proteins rise with the malignancy of the gliomas. Further investigations must be made to elucidate the relation between TERT and all GABP proteins as it may play a key role in the gliomagenesis.

## INTRODUCTION

Gliomas are the most common primary tumors of the central nervous system (CNS). They comprise the diffuse astrocytomas and oligodendrogliomas World Health Organization (WHO) grade II, the anaplastic astrocytomas and oligondendrogliomas WHO grade III and also the most frequent one, the glioblastoma multiforme (GBM) WHO grade IV [1]. Primary glioblastomas (prim. GBM) and secondary glioblastomas (sec. GBM) differ from each other in the frequency (95% vs. 5%), mean age of diagnosis (62 years vs. 45 years), the time of clinical duration (6.3 vs. 16.8 months) and the median overall survival with treatment (11.3 vs. 27.1 months) suggesting that those two tumor entities are evolving from diverse precursor, neural cells [2–5]. The different approaches of treatment according to grading and clinical presentation of gliomas extent from gross total resection to radiotherapy of 50–60 Gy in concomitance and maintenance with temozolomide as chemotherapy [6]. While elderly patients over 70 years can profit from hypofractionated radiotherapy alone [7], no specific treatment has been presented for recurrent and secondary GBMs so far [8]. Many efforts have been made to standardize the treatment of each glioma but still with disappointing outcome of the prognosis.

The need of further understanding and classifying the gliomas led to an emerging of molecular markers. One marker that has been introduced is telomerase. Telomerase reverse transcriptase (TERT) as the catalytic subunit which together with an RNA template prolong the telomeres adding hexameric 5′ -TTAGGG- 3′ repeats on the end of the chromosomes [9]. This function is limited in normal somatic cells and after each divisions the telomeres are being shortened whereas cancer cells have an increased telomerase activity contributing to the unrestricted elongation of the telomeres and immortalization of the cell [10]. One of the mechanisms affecting the upregulation of telomerase are the mutational alterations of the promoter region of TERT (pTERT) and its transcriptional regulations [11]. Two most common mutations in the pTERT are the C228T (1,295,228, C>T) and C250T (1,295,250, C>T) which are located at -124 base pairs (bp) and -146 bp upstream of the TERT ATG start codon, respectively [12]. These two point mutations occur frequently in tumor cells that do not need a continual regeneration such as melanomas and gliomas [13]. Among the gliomas, 83% of the primary glioblastomas harbor those hotspot mutations with a different distribution between C228T and C250T (73% vs. 27%) while in lower glioma grades they seem to be rare [13, 14]. In this way, the mutant promoter status correlates with the elevated mRNA levels of TERT and therefore with the increased telomerase activation [15]. Both non-coding mutations at the promoter generate a purine-rich GGAAG binding site for the transcription factor GA-binding protein (GABP) of the E-twenty-six (Ets) family [16]. It has been shown, that the GABP is recruited to these mutations, thus binding and activating the pTERT in glioblastomas [17]. GABP reveals its full function as a heterodimeric- or tetrameric complex consisting of the GABPA, as the DNA binding subunit, and the GABPB with the actual transcription activity [18, 19]. GABPB subunit is encoded by two distinct genes: GABPB1 gene encodes the GABPB1 with its isoforms GABPB1-longer (GABPB1-L) and GABPB1-shorter (GABPB1-S) as the products of a different mRNA splicing whereas GABPB2 is encoded by the GABPB2 gene as a single isoform [20]. While all of them can dimerize with GABPA only GABPB1, GABPB1L and GABPB2 can homodimerize and create a tetramer of two GABPA/B heterodimers due to an leucine-zipper-like domain (LZD) at the C-terminus end [21]. Recent studies showed that the GABP tetramer forming isoforms, especially GABPB1-L, activate the mutant TERT promoter and a disruption of B1L generates telomeric loss in glioblastoma cell lines introducing the importance of GABPA/B isoforms in the mutated TERT promoter dependent gliomas [22].

Consequently, in this study we investigated the mRNA expression level of TERT and all GABPA/B isoforms and their correlation and interplay in the grade II, grade III gliomas as well as in the primary and secondary glioblastomas to understand their role in the gliomagenesis.

## RESULTS

**Figure 1:**
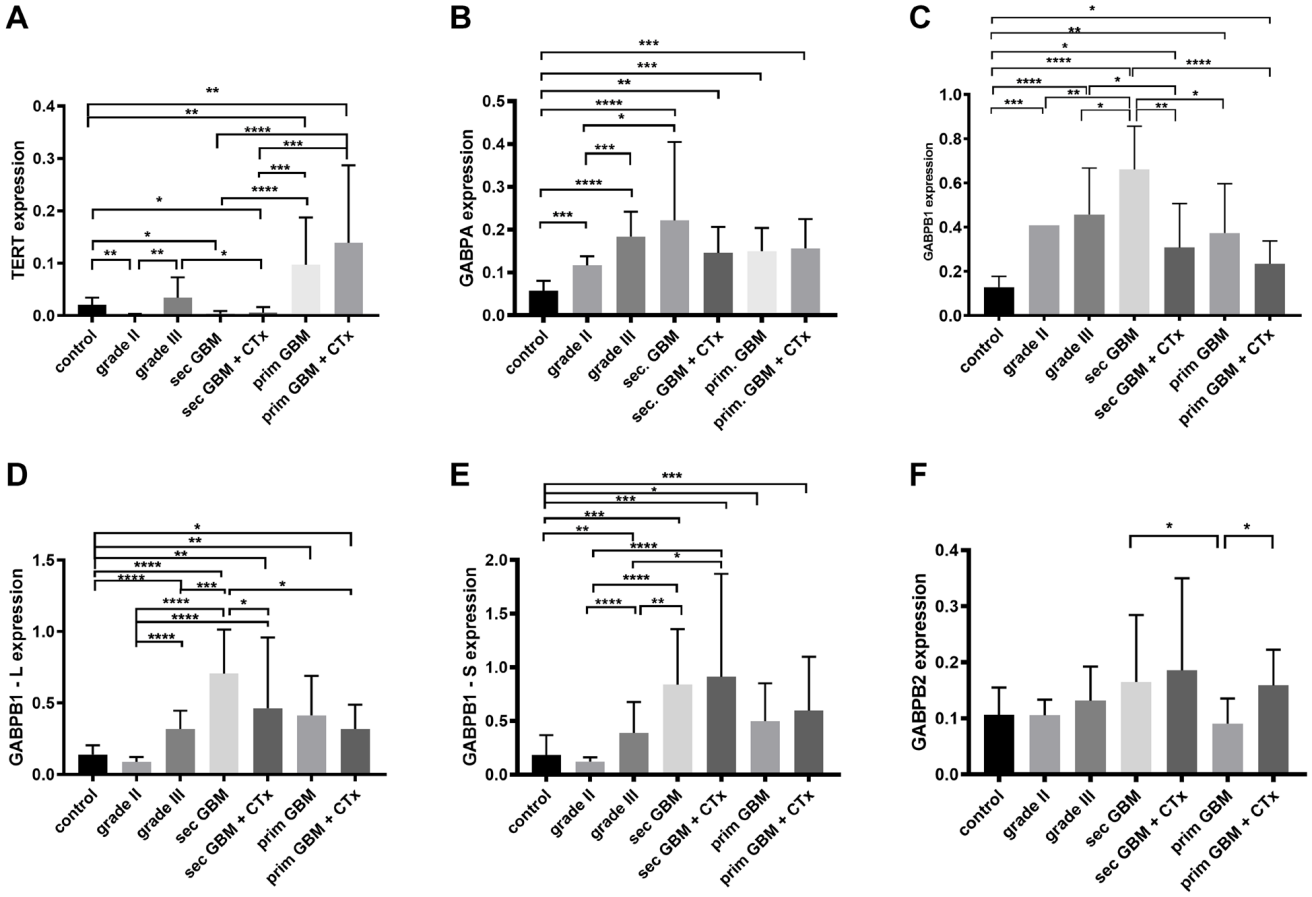
mRNA expression levels of (**A**) TERT, (**B**) GABPA, (**C**) GABPB1, (**D**) GABPB1–L, (**E**) GABPB1–S and (**F**) GABPB2 by quantitative real – time PCR in controls, glioma grade II, grade III, secondary glioblastomas (sec. GBM), recurrent secondary glioblastomas treated with chemotherapy (sec. GBM + CTx), primary glioblastoma (prim. GBM) and primary glioblastoma treated with chemotherapy (prim. GBM + CTx). Columns display the mean values [arbitrary units] and error bars the standard deviations. Statistically significance is marked with ^*^ *p* < 0.05, ^**^ *p* < 0.01, ^***^ *p* < 0.001 and ^****^ *p* < 0.0001.

We collected 70 tumor samples and analyzed their expression levels in control, grade II gliomas, grade III, secondary glioblastoma (sec. GBM), secondary glioblastoma with chemotherapy (sec. GBM + CTx), primary glioblastoma (prim. GBM) and primary glioblastoma with chemotherapy (prim. GBM + CTx). Each group consisted of 10 tumor samples. Figure 1 summarizes the expression of each protein in the different glioma grades.

### TERT in gliomas

Regarding the quantitative real-time PCR results and statistical analysis of our glioma cohorts we show that TERT mRNA is mostly expressed in primary GBM (mean = 0.097, 95% CI = 0.02–0.16) and in primary GBM which had been treated with chemotherapy (mean = 0.139, 95% CI = 0.02–0.25) compared to the control group (mean = 0.02, 95% CI = 0.01–0.03) with *p* = 0.007, respectively, without any difference between the two prim. GBM groups. Low grade gliomas, *p* = 0.003, sec. GBM with, *p* = 0.03, and without chemotherapy, *p* = 0.01, exhibit the lowest TERT levels compared to the normal brain tissue. Grade III gliomas express upregulation of TERT when compared to grade II, *p* = 0.009, without any significance to the peritumoral tissue. When primary and secondary glioblastomas each with different therapy status were compared, the results show significant elevation in both prim. GBM groups (prim. GBM vs. sec. GBM and sec. GBM + CTx: *p* < 0.0001 and *p* = 0.0002, respectively, prim. GBM + CTx vs. sec. GBM and sec. GBM + CTx: *p* < 0.0001 and *p* = 0.002, respectively) (Figure 1A).

### GABPA, GABPB1, GABPB1-L, GABPB1-S and GABPB2 in gliomas

We further analyzed all GABP components expression in the different glioma grades. The results indicate that GABPA, -B1, -B1-L and -B1-S have the trend to progress with the malignancy of the gliomas. Specifically, GABPA is being gradually expressed from control (mean = 0.057, 95% CI = 0.03–0.07) to grade II (mean = 0.116, 95% CI = 0.1–0.3, *p* = 0.0002), to grade III (mean = 0.184, 95% CI = 0.15–021, *p* = 0.0004) which is elevated compared to the control with *p* < 0.0001. GABPB1 seems to follow the same pattern of expression with grade II (mean = 0.409, 95% CI = 0.07–0.74) and III (mean = 0.456, 95% CI = 0.35–0.55) having high levels as to the peritumoral tissue (mean = 0.128, 95% CI = 0.09–0.16), *p* = 0.001 and *p* < 0.0001, respectively, but with no difference between them. GABPB1-L and -B1-S rise from grade II to grade III, both with *p* < 0.0001, and in grade III they are overexpressed compared to the non-tumorous tissue with *p* < 0.0001 and *p* = 0.004. GABPB1, -B1L and -B1S expression is progressing from grade III to sec. GBM with *p* = 0.016, *p* = 0.0009 and *p* = 0.002. All the GABPA/B isoforms express the highest levels in the secondary glioblastomas (GABPA: mean = 0.221, 95% CI = 0.09–0.35, *p* < 0.0001; GABPB1: mean = 0.66, 95% CI = 0.52–0.8, *p* < 0.0001; GABPB1-L: mean = 0.705, 95% CI = 0.48–0.92, *p* < 0.0001 and GABPB1-S: mean = 0.836, 95% CI = 0.46–1.2, *p* = 0.0004). In sec. GBM with chemotherapy treatment GABPB1 and -B1-L levels are downregulated (GABPB1: mean = 0.308, 95% CI = 0.16–0.45, *p* = 0.001 and -B1-L: mean = 0.464, 95% CI = 0.1–0.81, *p* = 0.035) as to sec. GBM and overexpressed compared to the control with *p* = 0.01 and *p* = 0.035, respectively. GABPA and -B1-S are also overexpressed when sec. GBM with chemotherapy and peritumoral tissue are compared, with *p* = 0.001 and *p* = 0.0006, but without any significant difference among the two sec. GBM groups. In primary glioblastomas with and without chemotherapy GABPA, -B1, -B1-L and -B1-S are overexpressed as to the free of tumor cells group (prim. GBM and prim. GBM + CTx in GABPA: *p* = 0.0002 and *p* = 0.0006; GABPB1: *p* = 0.001 and *p* = 0.035; GABPB1-L: *p* = 0.001 and *p* = 0.043; GABPB1-S: *p* = 0.027 and *p* = 0.001). GABPB2 did not show any statistically significant results in the gliomas. Figure 1B–1F shows the expression levels of GABPA/B isoforms and their relation between the glioma grades.

**Figure 2:**
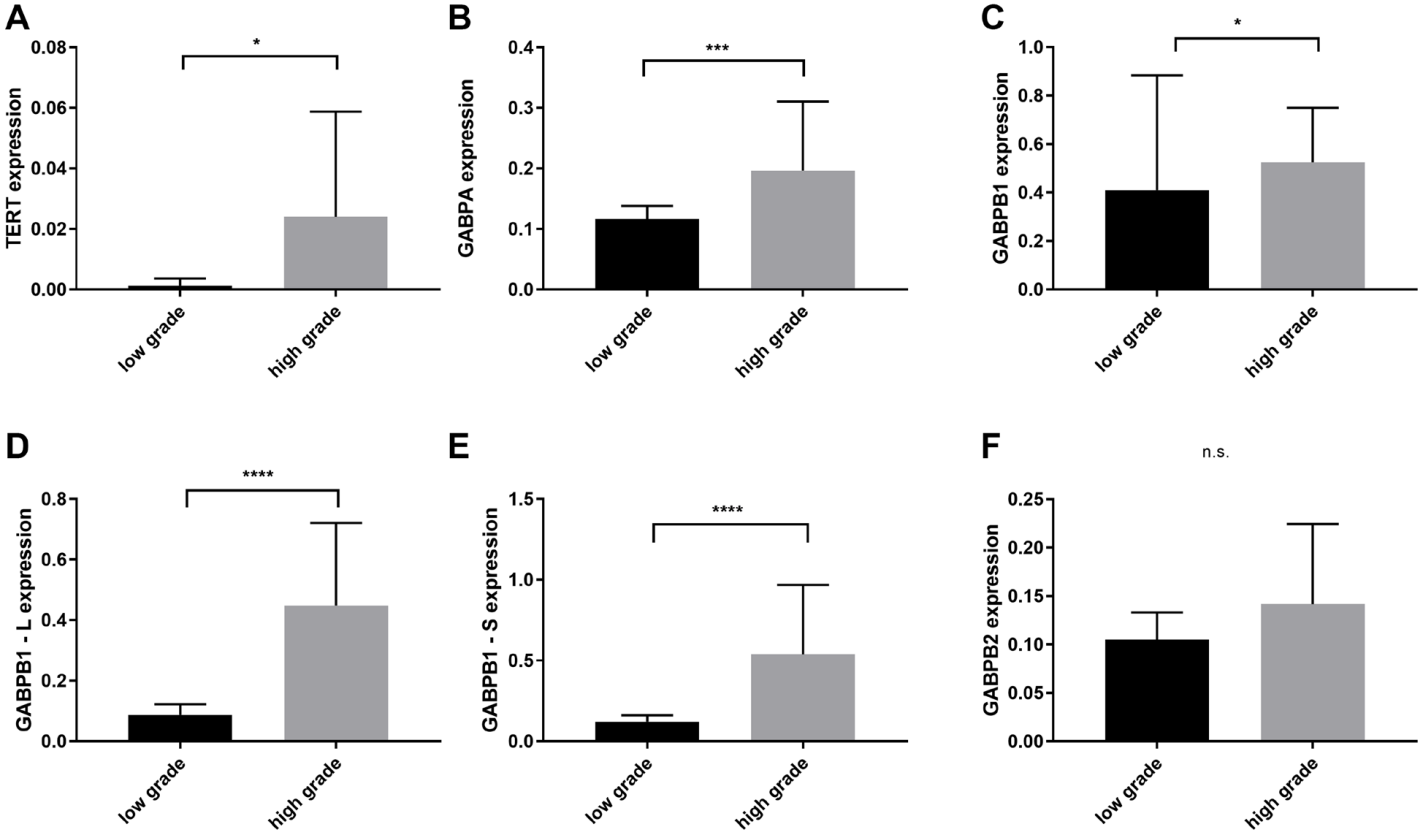
Comparison of TERT, GAPBA, GAPB1, GABPB1–L, GABPB1–S and GABPB2 mRNA expression levels between low grade (WHO II) and high grade gliomas (WHO III and secondary glioblastomas). (**A**) Overexpression of TERT in high grade gliomas. (**B**–**E**) Increased levels of GABPA, GAPB1, GABPB1–L, GABPB1–S in high grades. (**F**) GABPB2 shows no significant difference. Columns display the mean values and error bars the standard deviation. Statistical significance is marked with ^*^ *p* < 0.05, ^***^ *p* < 0.001 and ^****^ *p* < 0.0001, n.s.: no significance.

Subgrouping the different glioma groups in low and high grade gliomas (LGG and HGGs) consisting of grade III and secondary glioblastomas without chemotherapy, not only TERT but also GABPA, -B1, -B1-L and -B1-S were overexpressed in the HGGs (TERT: mean = 0.023, 95% CI = 0.01–0.03, *p* = 0.023, GABPA: mean = 0.196, 95% CI = 0.15–0.23, *p* = 0.0005, GABPB1: mean = 0.524, 95% CI = 0.44–0.6, *p* = 0.016, GABPB1-L: mean = 0.448, 95% CI = 0.34–0.55, *p* < 0.0001 and GABPB1-S: mean = 0.538, 95% CI = 0.37–0.69, *p* < 0.0001) (Figure 2).

**Figure 3:**
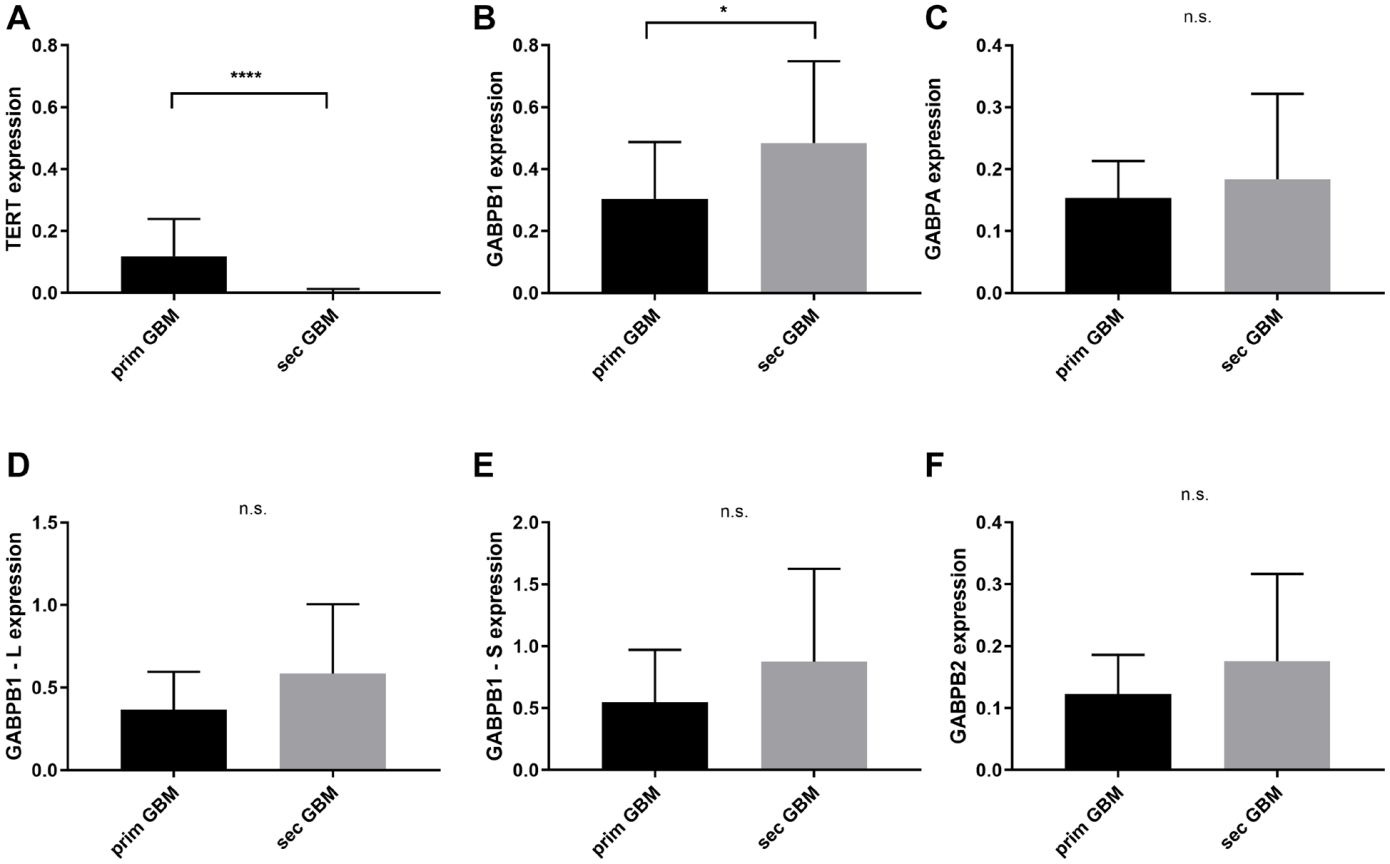
TERT and GABPA/B isoforms mRNA expressional status between primary and secondary glioblastomas with and without chemotherapy. (**A**) TERT is overexpressed in primary glioblastomas with *p* < 0.0001. (**B**) GABPB1 is highly expressed in secondary glioblastomas, *p* = 0.043. (**C**–**F**) No significant difference in GABPA, GABPB1–L, GABPB1–S and GABPB2. Columns display the mean values and error bars the standard deviation. Statistical significance is marked with ^*^ *p* < 0.05 and ^****^ *p* < 0.0001, n.s.: no significance.

Between the primary and secondary glioblastomas with and without chemotherapy treatment, TERT is significantly elevated in the prim. GBMs (mean = 0.118, 95% CI = 0.05–0.17) whereas GABPB1 is overexpressed in the sec. GBMs of the same therapeutical approach (mean = 0.484, 95% CI = 0.36–06) with *p* < 0.0001 and *p* = 0.043, respectively. The other transcription factors did not show any significant results when these two groups where compared. GABPB2 mRNA status in both sub-groups did not show any significant results (Figure 3).

### Correlation of TERT and GABP A/B isoforms in gliomas

**Figure 4:**
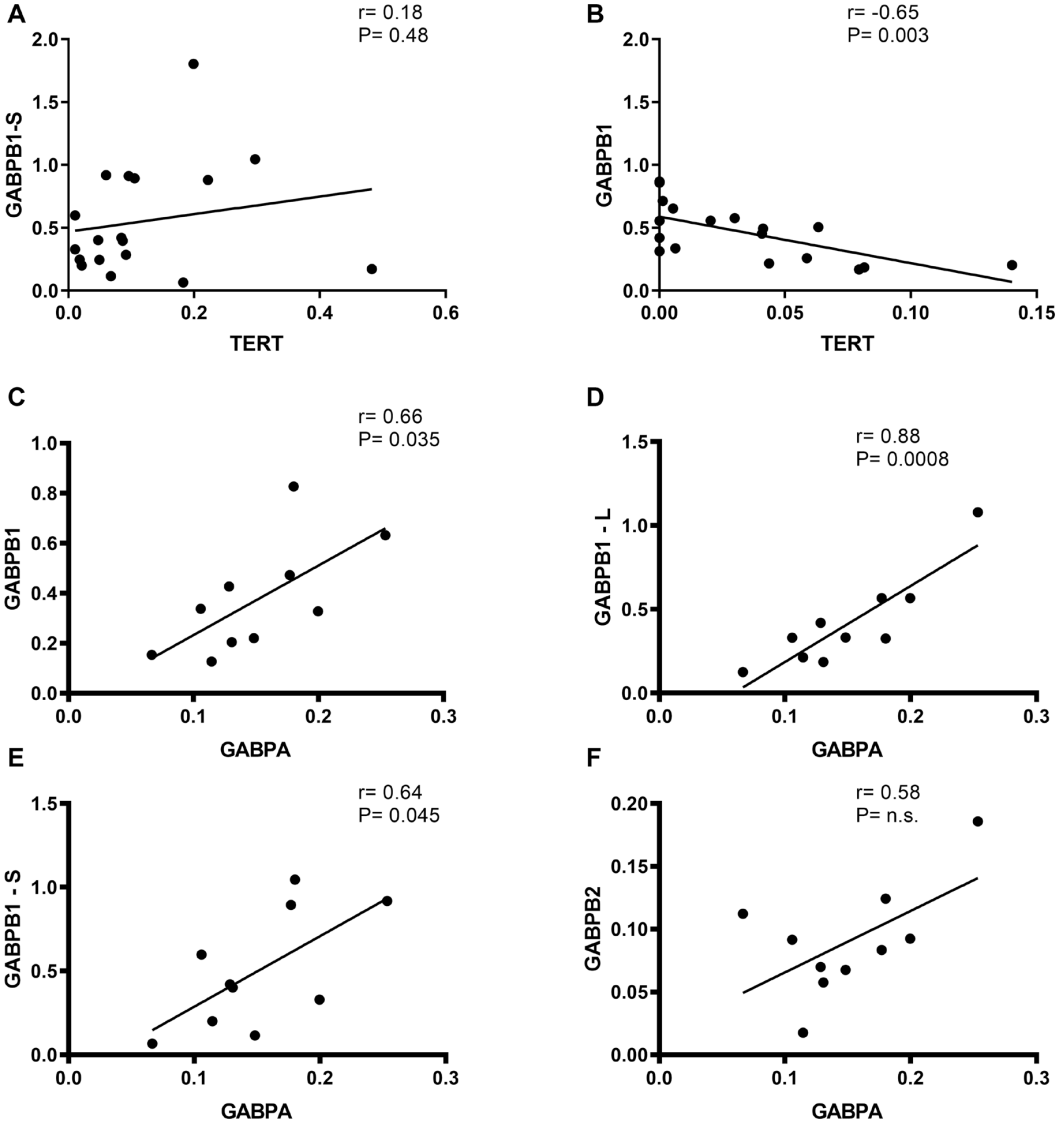
Correlation of TERT vs. GABPB1/-S and GABPB1, GABPB1-L, GABPB1-S and GABPB2 vs. GABPBA mRNA expression in gliomas. Black line indicates trend-line and Pearson’s rank order correlation was used to generate Pearson rho and *p-value*s for each correlation. (**A**) No correlation between TERT and moreover, in primary glioblastomas (*r* = 0.18). (**B**) negative linear correlation between TERT and in addition, in grade III gliomas (*r* = –0.65, *p* = 0.003). (**C**–**E**) GABPA correlates positive with GABPB1, GABPB1-L and GABPB1-S in primary glioblastomas with *p* = 0.035, 0.0008 and 0.045, respectively. (**F**) GABPA and GABPB2 have the tendency to slightly positive correlate in primary glioblastomas, without any statistical significance, n.s.: no significance.

Analyzing the association between TERT and each GABP component, we found that GABPB1-S did not show a correlation with TERT in primary glioblastomas with and without chemotherapy treatment (*r* = 0.18, 95% CI = −0.3–0.6; *p* = 0.48). In grade III gliomas, GABPB1 negative correlates with TERT significantly (*r* = −0.65, 95% CI = −0.85–0.27; *p* = 0.003). We further examined the relation between GABPA and every GABPB isoform. We found positive correlated relationship between GABPA and GABPB1 (*r* = 0.6, 95% CI = 0.06–0.91; *p* = 0.035), GABPB1-L (*r* = 0, 8, 95% CI = 0.56–0.97; *p* = 0.0008) and GABPB1-S (*r* = 0.6, 95% CI = 0.02–0.9; *p* = 0.045) in primary glioblastomas (Figure 4).

## DISCUSSION

Cancer cells can replicate continuously overcoming normal cell death. One of the mechanisms contributing in immortalization and limitless progression is the maintenance of the telomere endings of the chromosomes. Most of the malignant cells achieve it through upregulation of telomerase and fewer by an alternative lengthening of telomeres (ALT) [25]. The expression of TERT can be regulated by many molecular mechanisms such as transacting factors, epigenetic modifications and genetic promoter alterations which affect the activation of telomerase [26]. In this study, we presented that TERT mRNA expression levels are the highest in the primary glioblastomas compared to normal brain tissue. Similar data, regarding TERT gene expression on mRNA in prim. GBM have been previously described highlighting the positive association between expressional status and telomerase activity and suggesting that the promoter mutations are the major regulators of TERT expression [15, 27–29]. On the other hand, a common single nucleotide polymorphism, rs2853669 A>C, located at -246 bp upstream of the ATG start codon within a preexisting Ets2 binding site of the promoter which can be found in numerous GBMs, seems to cause a two-fold reduction of TERT transcription by disrupting the binding position [31, 32]. Both regulatory pathways have an impact on survival status where promoter mutations reduce overall survival compared to wild type (median 11–12 vs. 20 months) while in co-existence with the C-variant of rs2853669, the survival is shortened to 8–12 months [30]. In addition, the recurrent prim. GBMs of our cohort which have undergone chemotherapy, in most cases temozolomide, showed also elevated TERT levels. This data could provide proof of persisting TERT and other genomic alterations from primary to recurrent GBM as well as unstoppable cellular replication through telomere maintenance [10, 33]. Moreover, the transcription of TERT in a cohort of low grade gliomas and sec. GBM showed significant downregulation whereas grade III gliomas did not present any difference compared to the control. This could be explained due to the fewer amount of grade II (44%), III gliomas (60%) and sec. GBMs (28%) harboring TERT promoter mutations and therefore decreased transcriptional activation [14, 34, 35].

As we described before, the two somatic mutations in the promoter region of TERT create a *de novo* binding motif for GABP [16]. Analysis of the GABP structure reveals a multimeric complex consisting of GABPA and GABPB, two different proteins with different function, but both necessary components to create a functional, unique among the Ets family, transcription factor [18]. GABP as a tetrameric protein has been found to regulate lineage-restricted genes well defined in myeloid cells [36], to participate in cell cycle control while both molecule components can be found widely expressed in liver, muscle and brain cells [37]. The proof, that GABP is recruited to the hotspot mutations of the promoter, thus reactivating and inducing TERT expression in glioblastoma cell lines, gave a significant role to GABP as an transcription regulator in a TERT dependent manner and provides evidence of specific cancer interaction in the promoter core which may lead to limitless replication [17]. Many molecular alterations have been found to occur during gliomagenesis and glioma progression. Isocitrate dehydrogenase 1/2 (IDH1/2) mutations occur at 70% of grade II and III gliomas as an early event with better prognostic survival [3]. Secondary GBMs seem to have more than 80% of IDH1 mutations indicating the progression from diffuse gliomas and primary GBMs harbor fewer than 5% of IDH1 missense mutations and are identified as *de novo* tumors [5]. TP53 mutations in 94% and loss of α-thalassemia/mental-retardation-syndrome-X-linked (ATRX) in 33% of IDH mutant astrocytomas grade II/III and 1p/19q codeletions with IDH mutations classify the diffuse and anaplastic oligodendrogliomas [38–40]. We are the first to present an upregulation of all GABP components in the different glioma grades and GABPA, -B1, -B1-L, -B1-S are gradually expressed during malignancy progression from lower to higher grade while the most expression is observed in the sec. GBMs. This data may suggest that the different GABP proteins could be new glioma specific markers of carcinogenesis. GABPB isoforms can heterodimerize with GABPA and function as dimers while all component interact with each other and can be modulated by different proteins which could affect their expression [36]. This can be confirmed also by our studies where the expression of GAPBA and GABPB isoforms positive correlates in the prim. GBMs. Furthermore, GABPA/B1, B1-L, B1-S proteins in the primary GBMs of our cohort exhibit a significant upregulation compared to the normal brain tissue which can confirm at some point the finding of Mancini et al. He proved, that the B1L isoform is the main regulator of TERT expression in promoter mutated glioblastomas and there is a positive association between TERT and B1L mRNA expression, unlike our correlation results [22]. At this point it should be mentioned, that we did not investigate the samples for the mutational status of TERT promoter. Also, another important finding of Mancini and his group was that siRNA inhibition of B1L leads to reduction of TERT expression, thus inducing telomeric loss and cellular death [22]. Our findings propose that chemotherapy treatment in sec. GBMs decreases GABPB1 and GABPB1L mRNA levels and it could be used as a potential therapeutical approach together with targeting therapy in TERT and GABP dependent glioblastomas.

## MATERIALS AND METHODS

### Patients

For this study we obtained the surgical specimen from 80 patients that had been treated at the Department of Neurosurgery of the University Hospital of Cologne from 1990 to 2012. We analyzed 70 tumor tissue samples altogether. Ten patients were followed for progression during the disease. Each sample was than histopathologically graded according to the WHO classification of the CNS tumors 2016 [23] from two independent neuropathologists. All patients gave their informed written consent to use the samples as stated by the Declaration of Helsinki and the study was approved by the local ethic committee of the University of Cologne (Application No. 03-170).

### Glioma samples

The tumor samples were extracted during neurosurgery and snap-frozen in liquid nitrogen at −80°C and stored at our tumor bank after the neuropathologist’s examination and prior to the RNA isolation. Ten micrometers cryostat sections were taken of each sample and stained with hematoxylin & eosin. Each group consists of ten tissue samples and peritumoral tissue free of tumoral cells was used as the control.

### RNA extraction, cDNA synthesis and primers

RNA was isolated from the fresh frozen tissue using the RNeasy Mini Kit (Qiagen, Hilden, Germany). The quantity and purity of the isolated RNA was assessed spectrophotometrically at 260 and 280 nm. The cDNA-synthetization was performed with QuantiTect Reverse Transcription Kit (Qiagen, Hilden, Germany). The primers for telomerase reverse transcriptase (TERT) and the GA-binding protein A and B isoforms (GABPA/B isoforms) were designed and purchased from Eurofins, Genomics (Ebensberg, Germany). We used the succinate dehydrogenase complex, subunit A, flavoprotein variant (SDHA) as the housekeeping gene [24] (QuantiTect PrimerAssay; Qiagen, Hilden, Germany) (Table 1).

**Table 1:** Oligosequences for primers

Protein	Oligoname	Function	Sequence 5′→3′ (Forward)	Sequence 3′→5′ (Reverse)
Telomerase reverse transcriptase	TERT	Catalytic enzyme of telomerase	CGGCGACATGGAGAACAAG	CCAACAAGAAATCATCCACCAAA
GA-binding protein A	GABPA	Transcription factor	CCTGAACTGGTTGCACAGAA	ACAAATCATGTCCCCATCG
GA-binding protein B1	GABPB1	Transcription factor	GGCTGAAGCGCTAGAAATGG	GGAGAGAGGGGAAAGAGGGT
GA-binding protein B1 Long	GABPB1-L	Transcription factor isoform	AACCAGTGGAATTGGTCAGC	TGTAGGCCTCTGCTTCCTGT
GA-binding protein B1 Short	GABPB1-S	Transcription factor isoform	AACCAGTGGAATTGGTCAGC	ACCGGGTAAAAGACTCCTTAC
GA-binding protein B2	GABPB2	Transcription factor	AGCAAGTAATGGGGAGTGGA	AACCTTACCAGCAGGTACAG

### Quantitative real time-PCR

The quantitative real time polymerase chain reaction was done in triplicates. We used the final volume of 20 μl consisting of 1× Rotor Gene SYBR Green PCR Kit (Qiagen, Hilden, Germany), 10 μl Master Mix (Qiagen, Hilden, Germany), 5 μl of each primer and 5 μl of cDNA diluted in 1:50. The amplification was performed on a Rotor-Gene Q cycler (Qiagen, Hilden, Germany) in a two-step cycling protocol and the cycling conditions are the following: initial denaturation for 5 min in 95°C, followed by 95°C for 5 sec, followed by 60°C for 10 sec, each 35–45 cycles depending on the gene. After each run a melting curve was added.

### Statistical analysis

The analysis was performed using Graph Pad, Prism Version 8 (La Jolla, San Diego, California, USA). To determine the significant differences between the groups, we used the Kruskal-Wallis-Test as a non-parametric alternative to one-way Analysis of variance (ANOVA). Mann-Whitney-*U* Test was carried out to compare the mRNA expression levels between the different glioma groups and the control subjects by not normally distributed variables. To measure the linear relationship between TERT and GABPA/B isoforms, Pearson’s correlation was performed. *P-value* of 0.05 or smaller was set as the statistically significant level.

## CONCLUSIONS

In conclusion, the present study confirms the transcriptional status of TERT which is upregulated in the prim. GBMs while the other glioma grades exhibit no elevated activation. We further expanded our analysis by investigating all molecular components of GABP and proved their enhanced expression among the glioma grades which exhibit a similar pattern of activation. We therefore can propose that GABP might be a potential biomarker in glioma classification, but further investigations must be made in order to elucidate the actual relationship between TERT and GABPA/B isoforms in gliomas, aiming for targeted therapy in the future.

## Abbreviations

GBM: Glioblastoma
GII: WHO grade II
GIII: WHO grade III
GABP: GA-binding protein
Ets: E-twenty-six
OS: overall survival
PFS: progression-free survival
rt-PCR: real-time polymerase chain reaction
SDHA: Succinate dehydrogenase complex, subunit A, flavoprotein variant
TERT: Telomerase reverse transcriptase
WHO: World Health Organization
